# An Evaluation of the Proposed Worker Protection Standard with Respect to Pesticide Exposure and Parkinson’s Disease

**DOI:** 10.3390/ijerph14060640

**Published:** 2017-06-14

**Authors:** Alica Stubnova Sparling, David W. Martin, Lillian B. Posey

**Affiliations:** 1 Department of Economics, Davidson College, Davidson, NC 28035, USA; alsparling@davidson.edu; 2 Departments of Economics and Environmental Studies, Davidson College, Davidson, NC 28035, USA; 3 Metropolitan Housing and Communities Policy Center, Urban Institute, Washington, DC 20037, USA; poseylily@gmail.com

**Keywords:** Parkinson’s disease, pesticide exposure, benefit analysis, cost analysis

## Abstract

Citing a lack of information, the U.S. Environmental Protection Agency prudently did not account for the benefits of averting many chronic diseases in analyzing the Worker Protection Standards (WPS) revisions. We demonstrate that sufficient information can exist, using the example of the benefits to agricultural workers of reduced Parkinson’s disease (PD) due to reduced pesticide exposure. We define the benefits as the monetary value gained by improving quality of lives of people who would otherwise develop PD, plus the value of medical care cost averted and income not lost due to being healthy. For estimation, we use readily available parameters and obtain odds ratios of developing PD by conducting a meta-analysis of studies linking pesticide exposure to PD. The sensitivity analysis varies the number of agricultural workers affected by the regulation, the probability of being diagnosed with PD, the measurement and the timing of the benefits. Our initial assessment is that the reduced PD benefits would be a small fraction of the total WPS revision costs. However, if we define benefits as the common environmental economics willingness to pay to avoid PD incidence, then they become a substantial fraction of the costs. Our analysis demonstrates that the benefits of averting PD from the WPS revisions can be estimated using existing information, and that the results are most sensitive to the choice of valuation of benefits to the worker. We encourage other researchers to extend our framework to other chronic ailments.

## 1. Introduction

In August 2015, the U.S. Environmental Protection Agency (EPA) announced that the Worker Protection Standards had been revised [1]. The changes, which took effect on 2 January 2017, require increased information about, and training in, handling pesticides, posting notification signs for fields with recently applied pesticides, and ensuring that workers wear properly fitted protective equipment [2]. The EPA estimated that these protection efforts would likely cost $60.2–$66.9 million per year ([3], Lines 1218–1221). The Agency estimated that the readily measurable benefits would be only $0.6–$2.6 million per year (Table 2 in [3]). Critically, for our analysis, the EPA prudently excluded the benefits of avoiding incidents of such chronic ailments as non-Hodgkin’s lymphoma, prostate cancer, Parkinson’s disease, lung cancer, bronchitis, and asthma in its benefits estimation, because it felt that it lacked the appropriate information ([3], Section 4.7). While it did address those potential benefits in a supplementary break-even analysis ([3], Section 4.8), the possibility that relevant information to directly estimate those benefits does exist in the literature, motivated our analysis.

Our goals are (1) to demonstrate that the economic benefits of reducing chronic ailments with the WPS is feasible using the example of Parkinson’s disease (PD) and (2) to investigate the scale of those benefits. We use the example of PD because the relationship between PD and pesticide exposure has been of increasing interest to the public health community for some time [4,5,6,7]. Consequently, the development of an analytical framework that would allow for the explicit consideration of such an important health consideration for agricultural workers, is as important as the actual empirical values we estimate.

We estimate the total benefits of the new WPS as the monetary value gained by improving quality of lives of agricultural workers who would otherwise develop PD, plus the value of medical care cost averted and income not lost due to being healthy. We use readily available parameters in the literature for the estimation, and we conduct a meta-analysis of existing observational studies linking agricultural exposure to pesticides to onset of PD to obtain odds ratios of developing PD. We examine our results using sensitivity analysis, in which we vary the number of agricultural workers affected by the regulation, the probability of being diagnosed with PD, and the measurement and the timing of the benefits. 

We conclude first that our analytical framework is generalizable across a wide range of chronic ailments as it uses publicly available information for several parameters, and odds ratios from meta-analyses. Second, for the case of Parkinson’s disease, and with the aid of a sensitivity analysis that varies the number of agricultural workers affected by the regulation and the probability of being diagnosed, we conclude that the WPS would generate minimal economic benefits. However, thirdly, our sensitivity analysis allows us to conclude that the choice of how to measure the monetary benefits to a worker, matters greatly. If we use a methodology common to the health economics literature (the future benefits of improved quality of life without PD and the avoided medical and nonmedical costs associated with PD) we continue to observe minimal benefits. If, however, we use a methodology common to the environmental economics literature (the willingness to pay to avoid PD) then the estimated benefits increase substantially in magnitude. 

## 2. Methods

### 2.1. Deriving Benefits per Worker

The benefits from a regulation proposed to improve health, flow from two components: (1) the monetary value gained by improving the qualities of lives of people who would otherwise develop Parkinson’s disease (PD) and (2) the value of medical care costs not incurred, and income not lost, due to being healthy. We denote the regulatory benefits per worker as:

(1) $$\begin{array}{l} Benefits\;per\;Worker=P_{SPD|Exposure}\times \left(P_{Exposure|Old\;WPS}-P_{Exposure|New\;WPS}\right)\\ \;\;\;\;\;\;\times \sum_{t=PD\;onset}^{Death}\frac{\left[\left(QALY_{H}-QALY_{SPD}\right)V+\left(M_{SPD}-M_{H}\right)\right]}{{\left(1+r\right)}^{t}} \end{array}$$

where *P* is probability, *SPD* denotes that the worker suffers from Parkinson’s disease, *Exposure* denotes that the worker is exposed to chemicals that are linked to PD, *WPS* denotes Worker Protection Standard (the *Old* version that will be replaced by the *New* one), *H* denotes that the worker is healthy, *QALY* is one quality-adjusted life year, and *M* is the annual medical and non-medical (lost income) costs of illness. *V* is the monetary value we place on one *QALY*.

In Equation (1), the new WPS regulation lowers the probability of exposure to pesticides $\left(P_{Exposure|Old\;WPS}-P_{Exposure|New\;WPS}\right)$. We calculate the benefit per worker of the new WPS regulation by multiplying this decreased probability of exposure by the probability of developing PD if one is exposed to pesticides ($P_{SPD|Exposure})$ and by the monetary benefit of not having PD. The monetary benefit of not having PD is measured as the difference in the monetary value of quality of life with and without PD $\left((QALY_{H}-QALY_{SPD}\right)V)$ plus the difference in medical costs and income with and without PD $\left(M_{SPD}-M_{H}\right).$ Because a worker is expected to live for many years, the benefits of averting PD accrue over time (from the time of the onset of PD to death) and are discounted to the present at rate *r*.

### 2.2. Deriving the Probability Assessment in Terms of Odds Ratio (OR)

Our next step is to rewrite the probability of PD if one is exposed to pesticides ($P_{SPD|Exposure})$ in Equation (1) in terms of odds ratios (OR). The OR format is more practical for our aim to solve Equation (1) with available parameters because ORs are most commonly used for reporting results in the literature, both in observational studies and meta-analyses linking the relationship between pesticide exposure and PD. Odds ratios (OR) are typically given as in Equation (2).

(2) OR=PExposure|SPDPNo Exposure|SPDPExposure|Not SPDPNo Exposure|Not SPD

We rewrite the OR to reverse the conditional probabilities.

(3) OR=PExposure,SPDPNo Exposure,SPDPExposure,Not SPDPNo Exposure,Not SPD=PSPD|ExposurePSPD|No ExposurePNot SPD|ExposurePNot SPD|No Exposure

Then we manipulate this version of the OR to solve for $P_{Exposure|SPD}$.

$$OR\left(P_{Not\;SPD|Exposure}\right)=P_{SPD|Exposure}\left(\frac{P_{Not\;SPD|No\;Exposure}}{P_{SPD|No\;Exposure}}\right)$$

$$OR\left(1-P_{SPD|Exposure}\right)=P_{SPD|Exposure}\left(\frac{P_{Not\;SPD|No\;Exposure}}{P_{SPD|No\;Exposure}}\right)$$

(4) $$P_{SPD|Exposure}=\frac{OR}{\left(OR+\frac{P_{Not\;SPD|No\;Exposure}}{P_{SPD|No\;Exposure}}\right)}$$

Equation (4) can be written in terms of the OR and other readily available information if we make the simplifying assumption that the probability of suffering from Parkinson’s disease if you have not been exposed to the agricultural chemicals is the same as the overall probability of suffering from Parkinson’s disease. This assertion is based on the assumption that the number of agricultural workers (about 2 million ([3], Lines 68–71)) is a small proportion of the U.S. population (about 320 million [8]). Thus,

(5a) $$P_{SPD|No\;Exposure}\approx P_{SPD}$$

(5b) $$P_{Not\;SPD|No\;Exposure}\approx P_{Not\;SPD}=1-P_{SPD}$$

We can now rewrite the probability terms in Equation (4) as:

(6) $$P_{SPD|Exposure}=\frac{OR}{\left(OR+\frac{1-P_{SPD}}{P_{SPD}}\right)}$$

We can now rewrite Equation (1) expressing Benefits per Worker as:

(7) $$\begin{array}{l} Benefits\;per\;Worker\\ \;\;\;\;=\frac{OR}{\left(OR+\frac{1-P_{SPD}}{P_{SPD}}\right)}\times \left(P_{Exposure|\;Old\;WPS}-P_{Exposure|New\;WPS}\right)\\ \;\;\;\;\times \sum_{t=PD\;onset}^{Death}\frac{\left[\left(QALY_{H}-QALY_{SPD}\right)V+\left(M_{SPD}-M_{H}\right)\right]}{{\left(1+r\right)}^{t}} \end{array}$$

### 2.3. Deriving Total Benefits for All Agricultural Workers

Our next step is to account for the number of agricultural workers affected and to measure the benefits for 10 years in order to be consistent with the EPA’s time window of 10 years for assessing regulatory costs ([3], Line 1488). We denote those 10 years with the notation *i*.

(8) $$\begin{array}{l} Total\;Benefits\;for\;all\;agricultural\;workers\;affected\\ \;\;\;\;\;\;=\;\sum_{i=0}^{10}\frac{\#Workers\;\times \;Benefits\;per\;worker\;}{{\left(1+r\right)}^{i}} \end{array}$$

where Benefits per Worker are expressed in Equation (7). 

### 2.4. Using Available Parameters for Solution

Total benefits for all agricultural workers in Equation (8) can be solved using parameters readily available in the literature and by using ORs obtained from meta-analyses. Table 1 summarizes the ten parameters we take from the literature. In this section we describe the sources and steps taken to obtain these values. Then, in Equations (9) through (13) we demonstrate incorporating them into Equation (8). In Section 2.5 we discuss and demonstrate incorporating ORs into the analysis.

*QALY_H_* represents the Quality Adjusted Life Index in the cases where the worker is healthy and *QALY_SPD_* if the person suffers from PD. There are a variety of *QALY* estimates, both for healthy people and for people with PD [9,10,11,12]. We use the *QALY* for PD patients (*QALY_PD_* = 0.667) and the *QALY* for healthy people (*QALY_H_* = 0.810) estimated by Vossius et al. [13]. We use $100,000 as the value of one *QALY* gained (*V)* because Chandra et al. [14] noted that it is the value by which an effective regulation is measured.

We use O’Brien et al.’s [15] recent comprehensive assessment of $21,626 for the diverted medical and non-medical costs of PD, which includes $12,491 for the direct medical costs of PD (hospitalization, physician visits, medications and diagnostic tests) and $9135 for indirect costs of PD (lost wages of PD patient, lost wages of PD patient caregiver, personal care aide and death related costs). This estimate is not dissimilar from earlier values [16,17,18].

The U.S. Department of Agriculture estimated that the median age of the hired farm workers is 35 years, which we use as *t = 0* in our analysis [19]. We assume that the age of PD onset is 60, *t = 25* years from the current age of 35, which is consistent with the Michael J. Fox Foundation for Parkinson’s Disease Research statement [20] as well as previous studies [21,22,23,24,25,26,27]. We use age 80 (*t = 45*) as the age of death, as the life expectancy of 35 year olds is about 45 years from that age, across the range of reported genders and races in the National Vital Statistics Reports (Table 7 in [28]). Because it appears that PD does not shorten a person’s life [29,30], we use the same age of death for workers who remain healthy as for those with PD. We follow the EPA in using a 3% discount rate (*r*) ([3], Line 1220).

After incorporating those parameter estimates (numbers 1–7 in Table 1), we can compute the Benefits per Worker unadjusted for probabilities as:

(9) $$\overset{Death}{\underset{t=PD\;onset}{\sum }}\frac{\left[\left(QALY_{H}-QALY_{SPD}\right)V+\left(M_{SPD}-M_{H}\right)\right]}{{\left(1+r\right)}^{t}}=\overset{45}{\underset{t=25}{\sum }}\frac{\text{\$}35,926}{1.03^{t}}$$

The EPA estimated that the annual preventable and possibly preventable incidents rate as 0.05 per 1000 farmworkers due to the WPS (Table 4.5-3 in [3]), which we use as $\left(P_{Exposure|\;Old\;WPS}-P_{Exposure|New\;WPS}\right)$. Incorporating that value (parameter number 8 in Table 1) gives us:

(10) $$Benefits\;per\;Worker=P_{SPD|Exposure}\times \frac{0.05}{1000}\times \overset{45}{\underset{t=25}{\sum }}\frac{\text{\$}35,926}{1.03^{t}}$$

Our next step is to account for the number of agricultural workers affected, and to measure the benefits across 10 years, in order to be consistent with the EPA’s time window of 10 years for measuring costs. Interestingly, the number of workers that might be affected by the WPS is debatable. The EPA primarily used a value of about 2 million workers based upon the 2012 Census of Agriculture, although it also acknowledged the U.S. Department of Agriculture’s estimate of about 1 million workers ([3], Lines 68–71). In the text, we use the value of 2 million and include the 1 million value as part of our sensitivity analysis (parameter number 9 in Table 1). Since the EPA used a time window of ten years to assess regulatory costs ([3], Line 1488), we similarly measure the regulatory benefits for ten years. We denote those ten years with the notation *i*, which contrasts with the notation *t* that we relate to the age of agricultural workers. Thus, using all available parameters we can rewrite Equation (8) as:

(11) $$\begin{array}{l} Total\;Benefits\;for\;all\;agricultural\;workers\;affected\\ =\;\sum_{i=0}^{10}\frac{\#workers\times P_{SPD|Exposure}\left(P_{Exposure|\;Old\;WPS}-P_{Exposure|New\;WPS}\right)\left[\sum_{t=25}^{45}\frac{\$35,926}{1.03^{t}}\right]}{1.03^{i}} \end{array}$$

$$=\overset{10}{\underset{i=0}{\sum }}\frac{2,000,000\times P_{SPD|Exposure}\times \frac{0.05}{1000}\times \left[\sum_{t=25}^{45}\frac{\$35,926}{1.03^{t}}\right]\;}{1.03^{i}}$$

According to the Michael J. Fox Foundation, the likelihood of a 60-year old individual being diagnosed with Parkinson’s Disease is 1 in 100 [31], which we use for *P_SPD_* (parameter number 10 in Table 1). We can now express the probability of PD if exposed to pesticides ($P_{SPD|Exposure})$ in terms of ORs:

(12) $$P_{SPD|Exposure}=\frac{OR}{\left(OR+\frac{1-P_{SPD}}{P_{SPD}}\right)}=\frac{OR}{\left(OR+99\right)}$$

Finally, we are in a position to rewrite the benefits of the WPA regulation so that the only remaining unknown variable is the OR. Inserting Equation (12) into Equation (11) yields:

(13) $$Total\;Benefits=\overset{10}{\underset{i=0}{\sum }}\frac{27,243,298\;\frac{OR}{\left(OR+99\right)}}{1.03^{i}}$$

### 2.5. Estimating and Integrating the OR from a Meta-Analysis into the Total Benefits

Now that we have specified the total benefits of the WPA regulation so that the only unknown variable is the OR (Equation (13)), we need to estimate an appropriate OR to integrate into that measure. We conducted a meta-analysis of the literature relating agricultural exposure to pesticides to PD and estimated the OR. We followed the standard methodology for conducting meta-analyses, generally following the examples of Priyadarshi et al. [32,33], Van Maele-Fabry et al. [34], Allen and Levy [35], and Pezzoli and Cerada [36]. We reviewed the literature available through PubMed (accessed between January and March 2014) using the search criteria of “Parkinson”, “pesticide”, and “case control”; we rejected articles that were not in English, did not report OR, focused on non-human subjects, included other neurodegenerative diseases that overlap with PD, and/or focused on the genetic pathology of PD. If a study cohort was repeated then the more recent or more relevant article was chosen. Out of the 866 articles available on PubMed, we included the 22 articles listed in Table 2 [22,24,25,26,27,37,38,39,40,41,42,43,44,45,46,47,48,49,50,51,52,53] and used the results that controlled for the age, sex, and smoking behavior of the individuals.

As an initial attempt to address the likely heterogeneity across these 22 studies, we conducted three separate meta-analyses for these studies. Table 2 is organized to distinguish between these three categories. The first category includes all 22 studies listed in Table 2. The second category includes only the 11 studies in which cases and controls from the U.S. were used; these cases are identified in the second column of Table 2. The third category includes only the 12 studies that had samples of predominantly agricultural populations.

As these were all case-control studies, the relevant result from each study was the OR and the associated 95% confidence intervals (CI). Because of the variety in study designs and results, we used the OR for results presented as never/ever exposed for pesticides in general, because this result was most consistently used. The exception to this is where the study only included results for one type of pesticide [40,46,47] or subgroup of pesticide, such as herbicide [41,53] or insecticide [52]. Gatto et al. [51] presented incremental OR depending on the number of chemicals to which people were exposed, for which we used the estimate for more than 12 chemical groups. 

**Figure A1:**
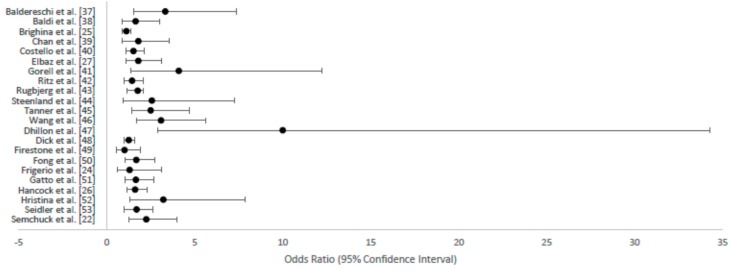
Forest Plot for All Included Studies.

**Figure A2:**
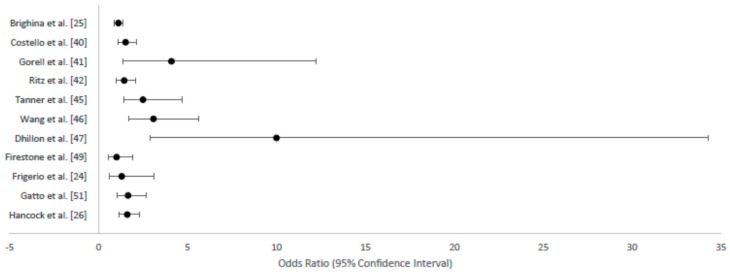
Forest Plot for Studies from the U.S.

**Figure A3:**
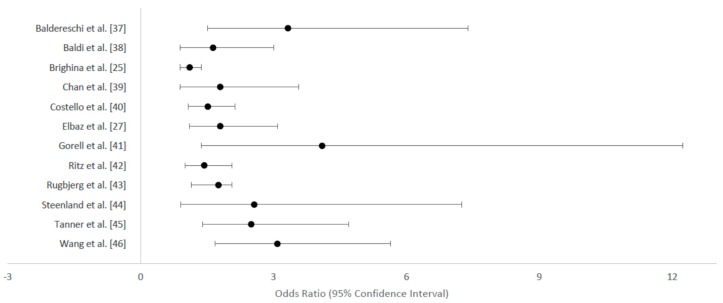
Forest Plot for Agricultural Studies.

Each of the five meta-analyses we cited earlier [32,33,34,35,36] found statistically significant levels of heterogeneity of studies, so it is not surprising that we also encountered a high degree of heterogeneity as measured by Cochran’s Q statistic [54]. The I^2^ statistic values confirm that over 50% of the variation in the estimates of the original treatment effects were due to heterogeneity [55], which is consistent with the three studies that reported I^2^ values. We illustrate that heterogeneity with forest plots in Appendix A (Figure A1, Figure A2 and Figure A3). We accounted for this bias by using the random effects (RE) model [55].

In Table 3 we present our meta-analysis results as well as those from the literature we cited earlier. Due to the congruence of these estimates, in this text we use the mean RE OR value of 1.81, which is the result using the subsample focusing on agricultural practices and the incidence of PD.

We can now insert the mean RE OR into Equation (13) and compute total benefits of the new WPS. For the mean RE OR of 1.81 from the subsample focusing on agricultural practices (see Table 3), the total benefits are $4,169,398, which we approximate as $4.2 million in our sensitivity analyses.

(14) $$\begin{array}{l} Total\;Benefits\;=\;\sum_{i=0}^{10}\frac{27,243,298\;\frac{OR}{\left(OR+99\right)}}{1.03^{i}}=\sum_{i=0}^{10}\frac{27,243,298\;\frac{1.81}{\left(1.81+99\right)}}{1.03^{i}}\\ \;\;\;\;\;\;\;\;\;\;\;\;=4,169,398 \end{array}$$

## 3. Results and Discussion

Instead of the 1.81 OR estimate that we used above, we begin our sensitivity analyses using the minimum value of the lower 95% CI ranges from Table 3 (1.03) as the estimate for OR. This lowest OR value gives us an estimate of the total benefits of the new WPS with respect to reducing pesticide-related PD incidence equal to $2.4 million. At the other extreme, the maximum OR value of the upper 95% CI ranges in Table 3 as the estimate for OR (2.60), we estimate that the total benefits of the new WPS with respect to reducing pesticide-related PD incidence could be as high as $5.9 million. These two values along with our original estimate of $4.2 million are presented in the middle columns of Panels A and B in Table 4 as well as in the first column of Panel C in Table 4.

### 3.1. Sensitivity Analysis 1: The Number of Affected Agricultural Workers

In Table 4, Panel A, we present the results of our first sensitivity analysis, the effects of halving and doubling both the number of agricultural workers affected by the regulation (halving corresponds to the U.S. Department of Agriculture’s estimate of the number of agricultural workers). This variable enters the benefits in a multiplicative manner (Equation (11)), so naturally halving and doubling the assumed values (which are in the center column) will halve and double the benefits estimate, respectively.

### 3.2. Sensitivity Analysis 2: The Probability of Being Diagnosed with PD

In Table 4, Panel B, we present the results of the second sensitivity analysis, which are the effects of halving and doubling the probability of being diagnosed with PD. As seen in Equation (12), this probability enters the benefits function in a non-linear manner. By coincidence the resulting benefits values are not dissimilar from the benefits values seen in Panel A.

### 3.3. Sensitivity Analysis 3: The Monetary Value of Avoiding PD

For our third sensitivity analysis, we explore the effects of the monetary value to a worker of avoiding PD. Our initial assessment, summarized in Equation (9) and based upon the standard health economics methodology, was that avoiding PD was worth $35,926 due to improved quality of life, and avoided medical costs and lost income. However, following the perspective common to environmental economics, the EPA used a Willingness to Pay (WTP) value of $1 million in their supplementary break-even analysis (which was based upon the WTP to avoid chronic bronchitis ([3], Lines 6220–6222)). Further, noting that PD shares some of the effects of peripheral neuropathy, the EPA determined that another viable WTP estimate could be $3.7 million ([3], Lines 6224–6234).

Incorporating this point into our analysis is not a straight-forward sensitivity analysis as the timing of this benefit does not occur as we modeled in Equation (1) from the onset of PD until death. Instead, given that the nature of WTP is to ask each worker for a present value assessment of avoiding PD, the correct valuation of the regulation’s benefits would be the following version of Equation (3), in which we assume that there are 2 million agricultural workers each year, and the regulatory benefits are only considered over 10 years.

(15) $$\begin{array}{l} Total\;Benefits\;\\ =\sum_{i=0}^{10}\frac{P_{SPD|Exposure}\left(P_{Exposure|\;Old\;WPS}-P_{Exposure|New\;WPS}\right)\left(2,000,000\right)WTP}{1.03^{i}}\\ =\sum_{i=0}^{10}\frac{\left(0.00005\right)\;\left(\frac{OR}{\left(OR+99\right)}\right)\left(2,000,000\right)WTP}{1.03^{i}} \end{array}$$

Our sensitivity results are presented in Table 4, Panel C. Clearly, this value is a critical one for the analysis. Using the OR value of 1.81, the initial benefits value of $4.2 million (using the $35,926 value gained by improving quality of life compared to PD, plus the value of medical care cost averted and income not lost due to being healthy) increases to $15.3 million (using the $1 million WTP value of the benefit per person) and then to $56.6 million (using the $3.7 million WTP-based value of the benefit per person).

### 3.4. Sensitivity Analysis 4: PD Onset Occurs Immediately

The importance of the timing of the benefits is emphasized by the results of the fourth sensitivity analysis in Table 4, Panel D. Here, we repeat the sensitivity analysis in Panel B but instead adjust the discounting so that the PD onset occurs immediately (*t = 0*) for the average worker instead of at age 60 (*t = 25*). These calculations result in increased benefits values that are higher than Panel B but are still substantially less than the values in Panel C. Thus, this unrealistic possibility emphasizes the large size of the impacts in Panel C, thereby illustrating the importance of choosing between the two approaches to estimating the health benefits of reducing PD incidence.

### 3.5. Discussion

Our initial estimate of the present value of the total benefits of averting PD ranged from $2.4 to $5.9 million, which shows only a relatively small beneficial contribution, given the annual costs of the proposal ($60.2–$66.9 million ([3], Lines 1218–1221)). That assessment held through sensitivity analyses in which we varied the number of affected agricultural workers and the risk of suffering PD. That qualitative assessment of the size of the benefits of averting PD held even when we implausibly assumed that PD onset begins immediately.

Yet, when we changed the definition of the benefits gained from averting PD we did see that the benefits increased substantially. In our original model, which yielded those low benefits, we based our analyses on the standard health economics concept of an improved *QALY* from averting PD, the value of that *QALY*, and the averted costs of illness. In contrast, when we used a framework common to environmental economics, the WTP to avoid PD, then the benefits more than tripled (as estimated by the WTP to avoid chronic bronchitis) or increased by more than an order of magnitude (as estimated by the WTP to avoid peripheral neuropathy). Thus, we show that the researchers’ choice of the approach to valuing avoiding PD, and indeed likely any chronic ailment, is the crucial step in the benefit analysis.

## 4. Conclusions

In this paper we presented an analytical framework for estimating total monetary benefit of averting PD thanks to the new WPS aimed to lower the exposure to pesticides by agricultural workers. Our paper was motivated by the EPA’s decision to estimate the benefits of the WPS revisions without including the benefits of avoiding PD and many other chronic ailments, citing a lack of information. However, in this paper we demonstrated that researchers can use existing studies on the association between pesticide exposure and onset of chronic ailments as a useful source for meta-analyses thereby allowing them to estimate benefits that would otherwise be ignored.

Our analytical approach took advantage of parameters readily available in the literature and of ORs obtained from meta-analysis that we conducted using existing observational studies. The framework we presented in this paper is generalizable. It can be used by researchers for estimation of benefits of averting other chronic ailments that similarly have estimates available for key parameters and meta-analyses (either available or possible to conduct) that link the onset of chronic ailments to agricultural exposure.

We agree with the EPA’s encouragement of further research to better explain the causal effect between pesticide exposure and chronic health outcomes including PD incidence ([3], Lines 5906–5908), as that research would provide more robust estimates of the required parameters. Additionally, we demonstrate that another valuable focus of future research would be on the methodology used to assess the benefits of averting chronic illnesses. The choice between a *QALY*-based benefit assessment and a WTP-based benefit assessment was clearly the most important aspect of the analysis. 

## Figures and Tables

**Table 1:** Parameters for Total Benefits.

Parameter		Value	Source
1.	*QALY_H_*	0.810	Vossius et al. [13]
2.	*QALY_SPD_*	0.667	
3.	*V*	$100,000	Chandra et al. [14]
4.	$M_{SPD}$	$12,491 + $9135	O’Brien et al. [15]
5.	*r*	3%	EPA ([3], Line 1220)
6.	*time = PD onset*	*t* = 25	Michael J. Fox Foundation for Parkinson’s Disease Research [20] and sources [21,22,23,24,25,26,27]
7.	*time = death*	*t* = 45	*National Vital Statistics Reports* (Table 7 in [28])
8.	$\begin{array}{l} P_{Exposure|\;Old\;WPS}\\ -P_{Exposure|New\;WPS} \end{array}$	$\frac{0.05}{1000}$	EPA (Table 4.5-3 in [3])
9.	*# Workers*	2 million	EPA ([3], Lines 68–71)
10.	*P_SPD_*	0.01	Michael J. Fox Foundation [31]

**Table 2:** Studies included in the Meta-Analysis.

Reference	Country	Cases (Exposed/Total)	Controls (Exposed/Total)	Odds Ratio	95% CI
*Studies that had samples of predominantly agricultural practices*					
Baldereschi et al. [37]	Italy	7/113	82/4383	3.33	1.51–7.39
Baldi et al. [38]	France	19/84	38/252	1.64	0.89–3.01
Brighina et al. [25]	USA	303/833	278/833	1.11	0.89–1.38
Chan et al. [39]	Hong Kong	19/215	16/313	1.80	0.90–3.58
Costello et al. [40]	USA	110/368	75/341	1.52	1.08–2.14
Elbaz et al. [27]	France	40/225	48/107	1.80	1.10–3.10
Gorell et al. [41]	USA	NA/144	NA/464	4.10	1.37–12.24
Ritz et al. [42]	USA	93/324	74/334	1.44	1.01–2.06
Rugbjerg et al. [43]	Canada	74/403	47/405	1.76	1.15–2.07
Steenland et al. [44]	Costa Rica	NA	NA	2.57	0.91–7.26
Tanner et al. [45]	USA	23/110	49/358	2.50	1.40–4.70
Wang et al. [46]	USA	46/362	18/341	3.09	1.69–5.64
*Other studies that met our inclusion criteria*					
Dhillon et al. [47]	USA	27/100	3.0/84	10.00	2.90–34.3
Dick et al. [48]	Scotland, Sweden, Italy, Malta, Romania	NA/767	NA/1989	1.25	0.97–1.61
Firestone et al. [49]	USA	19/156	28/241	1.01	0.53–1.92
Fong et al. [50]	Taiwan	85/153	66/155	1.68	1.03–2.76
Frigerio et al. [24]	USA	15/149	10/129	1.30	0.60–3.10
Gatto et al. [51]	USA	270/368	273/341	1.66	1.04–2.66
Hancock et al. [26]	USA	200/319	147/296	1.61	1.13–2.29
Hristina et al. [52]	Serbia	27/110	19/220	3.22	1.32–7.87
Seidler et al. [53]	Germany	59/380	44/379	1.70	1.00–2.60
Semchuck et al. [22]	Canada	32/130	30/260	2.25	1.27–3.99

**Table 3:** Meta-Analysis Results.

HeadlineMeta-Analysis		Cochran Q	I^2^	Lower	Mean	Upper
	N	Statistic *p*-Value	Statistic	95% CI	RE OR	95% CI
All included studies	22	*p* < 0.01	51.4%	1.51	1.74	2.00
Subsample of U.S. studies	11	*p* < 0.01	66.3%	1.35	1.73	2.21
Subsample of studies of agricultural practices	12	*p* < 0.01	56.4%	1.47	1.81	2.22
Priyardshi et al. [32]	19	*p* < 0.01	n.r.	1.49	1.94	2.53
Priyardshi et al. [33]	14	*p* = 0.01	n.r.	1.31	1.85	2.60
Van Maele-Fabry et al. [34]	12	*p* < 0.01	74.0%	1.03	1.28	1.59
Allen and Levy [35]	20	*p* = 0.03	40.0%	1.40	1.66	1.96
Pezzoli and Cereda [36]	51	*p* = 0.005	67.3%	1.56	1.76	2.04

**Table 4:** Sensitivity Analyses of Total Benefits of the new WPS for Agricultural Workers ($).

**Panel A: Sensitivity Analysis 1**				
		**Number of Agricultural Workers:**		
		1,000,000	2,000,000	4,000,000
Minimum OR	1.03	1,200,000	2,400,000	4,800,000
Agricultural Practices OR	1.81	2,100,000	4,200,000	8,300,000
Maximum OR	2.60	3,000,000	5,900,000	11,900,000
**Panel B: Sensitivity Analysis 2**				
		**Probability of Suffering PD**$\;\left(P_{SPD|No\;Exposure}\approx P_{SPD}\right)$:		
		0.005	0.010	0.020
Minimum OR	1.03	1,200,000	2,400,000	4,800,000
Agricultural Practices OR	1.81	2,100,000	4,200,000	8,300,000
Maximum OR	2.60	3,000,000	5,900,000	11,700,000
**Panel C: Sensitivity Analysis 3**				
		**Value of the benefit per person:**		
		35,925	1,000,000	3,700,000
Minimum OR	1.03	2,400,000	8,800,000	32,500,000
Agricultural Practices OR	1.81	4,200,000	15,300,000	56,600,000
Maximum OR	2.60	5,900,000	21,800,000	80,800,000
**Panel D: Sensitivity Analysis 4**				
		**Assume PD onset immediately instead of in 25 years** **Probability of Suffering PD** $\;\left(P_{SPD|No\;Exposure}\approx P_{SPD}\right)$:		
		0.005	0.010	0.020
Minimum OR	1.03	2,500,000	5,000,000	10,000,000
Agricultural Practices OR	1.81	4,400,000	8,700,000	17,300,000
Maximum OR	2.60	6,300,000	12,500,000	24,500,000

## Supplementary Materials

Supplementary File 1
